# Bayesian unsupervised clustering identifies clinically relevant osteosarcoma subtypes

**DOI:** 10.1093/bib/bbae665

**Published:** 2024-12-20

**Authors:** Sergio Llaneza-Lago, William D Fraser, Darrell Green

**Affiliations:** Biomedical Research Centre, Norwich Medical School, University of East Anglia, Norwich Research Park, Norwich NR4 7TJ, United Kingdom; Bioanalytical Facility, Norwich Medical School, University of East Anglia, Norwich Research Park, Norwich NR4 7UQ, United Kingdom; Biomedical Research Centre, Norwich Medical School, University of East Anglia, Norwich Research Park, Norwich NR4 7TJ, United Kingdom

**Keywords:** heterogeneity, latent process decomposition, osteosarcoma, precision medicine, RNA-seq

## Abstract

Identification of cancer subtypes is a critical step for developing precision medicine. Most cancer subtyping is based on the analysis of RNA sequencing (RNA-seq) data from patient cohorts using unsupervised machine learning methods such as hierarchical cluster analysis, but these computational approaches disregard the heterogeneous composition of individual cancer samples. Here, we used a more sophisticated unsupervised Bayesian model termed latent process decomposition (LPD), which handles individual cancer sample heterogeneity and deconvolutes the structure of transcriptome data to provide clinically relevant information. The work was performed on the pediatric tumor osteosarcoma, which is a prototypical model for a rare and heterogeneous cancer. The LPD model detected three osteosarcoma subtypes. The subtype with the poorest prognosis was validated using independent patient datasets. This new stratification framework will be important for more accurate diagnostic labeling, expediting precision medicine, and improving clinical trial success. Our results emphasize the importance of using more sophisticated machine learning approaches (and for teaching deep learning and artificial intelligence) for RNA-seq data analysis, which may assist drug targeting and clinical management.

## Introduction

Cancer treatment approaches (and drug licencing) are largely dictated by tissue site, but molecular profiling studies have shown that heterogeneity exists in the cancer driver gene landscape within and across tumor types [1]. A key objective of bioinformatics is to translate these comprehensive inventories of cell components and their mutations into mechanistic understanding and more accurate diagnostic labeling, leading to the development of stratified medicines and immunotherapies [2, 3]. Computational biological approaches, including hierarchical, k-means, and self-organizing clustering applied to cancer transcriptomes, have categorized clinically relevant breast cancer subtypes [4]. For rare cancers such as pediatric, adolescent, and young adult (AYA) cancers, no associations between molecular profiles, clinical presentation, or survival outcomes are known; therefore, untargeted chemotherapy remains the backbone of standard of care [5]. Pediatric and AYA cancers are clinically and biologically highly distinct from adult cancers, so bioinformatics methods need to be adapted and improved to make the best use of the few available samples.

Bone and soft tissue sarcomas account for ~1% of all cancer diagnoses [6] but combined are the third commonest pediatric and AYA cancer making up one in five cases [7]. Sarcomas are characterized by abnormal terminal differentiation [8–14] and genomes with complex structural rearrangements [7]. Osteosarcoma is the commonest bone sarcoma in the younger age group affecting ~3–4 per million individuals globally annually [6]. The disease typically originates in the metaphyseal intramedullary cavity of a long bone such as the femur, tibia, or humerus [15]. *TP53* or *RB1* loss-of-function or sometimes mutant gain-of-function [16, 17] is required for tumorigenesis [18]. This precursor cell does not enter apoptosis and instead undergoes further mutation including whole-genome doubling [19] causing disease progression and metastasis [20–22]. Osteosarcoma has one of the highest structural rearrangement rates of any cancer [23, 24]. This complex biology has routinely complicated discovery studies aiming to identify osteosarcoma subtypes.

A lack of identification of biological subgroups, understanding the role of the tumor immune microenvironment, factors that promote treatment resistance and metastasis plus identification of clinically relevant biomarkers of prognosis and drug response [3] means that the osteosarcoma 5-year survival rate has stagnated at ~50% for the last 45 years [6]. Phases I and II clinical trials investigating new medicines have not advanced to phase III [25–32]. The “failed” trials recruited patients with osteosarcoma as one entity but data mining shows that there was a small response rate (e.g., event-free, progression-free, etc.) in each trial (~5–15%). This small but importantly frequent response suggests that there are clinically relevant disease subtypes responsive to new therapies. The new medicines were not a total “failure” as was concluded; rather, the drugs were not successful for pan-osteosarcoma but could have become the standard of care for selected patient groups.

The prediction of osteosarcoma molecular subtypes has been tested using classical unsupervised learning methods. These studies support the existence of osteosarcoma subtypes [33, 34]. While original and important, a fundamental flaw to these computational approaches is that they inherently understate the heterogeneous composition of individual osteosarcoma tumors and assume sample assignment to a particular cluster. The analyses were performed in contrast to the well-reported heterogeneous components of most individual cancer samples [35–37]. Solid tumors are known to comprise different cell lineages that manifest as intratumour variation in transcriptomic output [9, 38].

Here, we used a more sophisticated unsupervised Bayesian method termed latent process decomposition (LPD) [39], which considers individual tumor sample heterogeneity. LPD is a Gaussian mixed membership model where the gene expression profile for a single sample is represented as a combination of the underlying latent (i.e., hidden) signatures with the combination weights drawn from a Dirichlet distribution. Each latent signature has a representative gene expression pattern. A given sample can be represented over a number of these underlying functional states or just one state. The appropriate number of signatures to use was determined by the LPD algorithm [35]. Grouping patients using this algorithm could provide clinical decision support.

## Methods

### Patients and datasets

We studied in silico data using four publicly available transcriptome datasets with primary osteosarcomas where the library preparation methods and sequencing parameters were not too dissimilar. These datasets were referred to as GREEN [40], PERRY [41], SCOTT [42], and Therapeutically Applicable Research to Generate Effective Treatments (TARGET) (https://www.cancer.gov/ccg/research/genome-sequencing/target) initiative phs000468. Patient and dataset characteristics including the sequencing platforms used and clinical data location are provided in Table 1. We retrieved the FASTQ files and performed quality control and trimming using TrimGalore (v0.6.5). Alignment was executed with HISAT2 (v2.1.0) against the reference human genome from ENSEMBL (hg38). The resultant BAM files were sorted and converted to SAM using SamTools (v1.11). Count matrices were generated using the R (v4.2.1) package Rsubread (v2.12.2). All processes were performed at the High Performance Computing (HPC) unit (https://www.uea.ac.uk/groups-and-centres/research-and-specialist-computing/high-performance-computing) at the University of East Anglia.

**Table 1:** Dataset characteristics. Overview of the RNA-seq datasets used in the study.

Dataset	Primary	Metastatic	Cell line	Circulating tumor cells	Normal Tissue	Platform	Citation
GREEN	7	3	0	5	0	Illumina HiSeq 2000	Green *et al.* [40]
PERRY	35	0	0	0	0	Illumina HiSeq 2000	Perry *et al.* [41]
SCOTT	35	9	5	0	3	Illumina HiSeq 2000	Scott *et al.* [42]
TARGET	88	0	0	0	0	Affymetrix Human Exon ST Array	phs000468
Total	165	12	5	5	3	Total samples: 190	

For each dataset, the number of samples across different sample types (e.g., primary tissue, metastatic tissue, circulating tumor cells, etc.) is provided. The sequencing platform used plus the citation of the original work is also included.

### LPD model development

LPD, a soft unsupervised Bayesian model, was used to classify samples into subgroups termed “processes”. For the input, we reduced the TARGET expression dataset to the top ~500 transcripts exhibiting the greatest variance (Supplementary File 1). The LPD model objectively assesses the most likely number of processes by tuning two hyperparameters: (i) the number of processes and (ii) the process spread (sigma). To achieve this step, we assessed the hold-out validation log-likelihood of the data at various combinations of the hyperparameters. The optimal combination number of signatures was identified as the point with the highest log-likelihood just before the overfitting region represented visually as a plateau. The hyperparameters determine the structure of the priors with the number of processes influencing the Dirichlet distribution for mixture weights and the sigma value affecting the Gaussian priors on gene expression variance. The mathematical formulas underlying the processes described above are reported [39]. To ensure robustness, LPD was performed 100 times with varying random seeds using the optimized parameters. Kaplan–Meier survival analysis and log-rank tests were conducted to identify runs yielding subgroups associated with a poor prognosis (e.g., low overall survival). The LPD run exhibiting the survival log-rank value closest to the mode was used for subsequent analyses.

### LPD model and dataset validation

The ~500 transcripts selected as the input for the TARGET dataset were also used as the input for the LPD model in the GREEN, PERRY, and SCOTT validation datasets. LPD was applied to each dataset separately using the same hyperparameter optimization described above. Due to the limited available clinical data in the GREEN, PERRY, and SCOTT datasets, the optimal LPD run was selected based on presenting a subtype closely resembling the TARGET-LPD “poor prognosis” subtype based on Pearson correlation of the median z-scores of the ~500 input transcripts. For these selected runs, clinical associations and sample type proportions were evaluated. For the PERRY dataset, Kaplan–Meier and Cox regressions were performed.

### Comparative analysis of LPD to traditional clustering methods

To assess the performance of LPD relative to traditional clustering methods, hierarchical clustering (with Ward’s D2 linkage) and k-means clustering (with Hartigan-Wong and 50 restarts) were applied to the TARGET dataset. The optimal number of clusters for each method was determined using the silhouette score. Kaplan–Meier survival curves were generated for the resulting clusters from each method to evaluate their ability to identify clinically relevant subtypes.

### Statistical analysis

All statistical tests were performed in R (v4.3.2). To characterize the identified processes, each sample was assigned to the process that had the highest degree of membership. ANOVA and Chi-square tests were used to compare sample type, age, ethnicity, and sex across groups (where available) using Bonferroni correction for multiple comparisons. Cox regression was used to determine the influence of process assignment on survival time. Differentially expressed (DE) transcripts for each process were identified using DESeq2 (v1.42.1) [43] with criteria set at absolute log_2_ fold change >1.5 and adjusted *p*-value <0.01 using Benjamin–Hochberg correction [44]. To ensure robustness, DE transcripts were only accepted if they were present in at least 80% of the poor prognoses runs. DE transcript functional analysis was performed using the Gene Ontology (GO) [45] database through clusterProfiler (v4.10.1) [46].

## Results

### Bayesian unsupervised clustering

**Figure 1:**
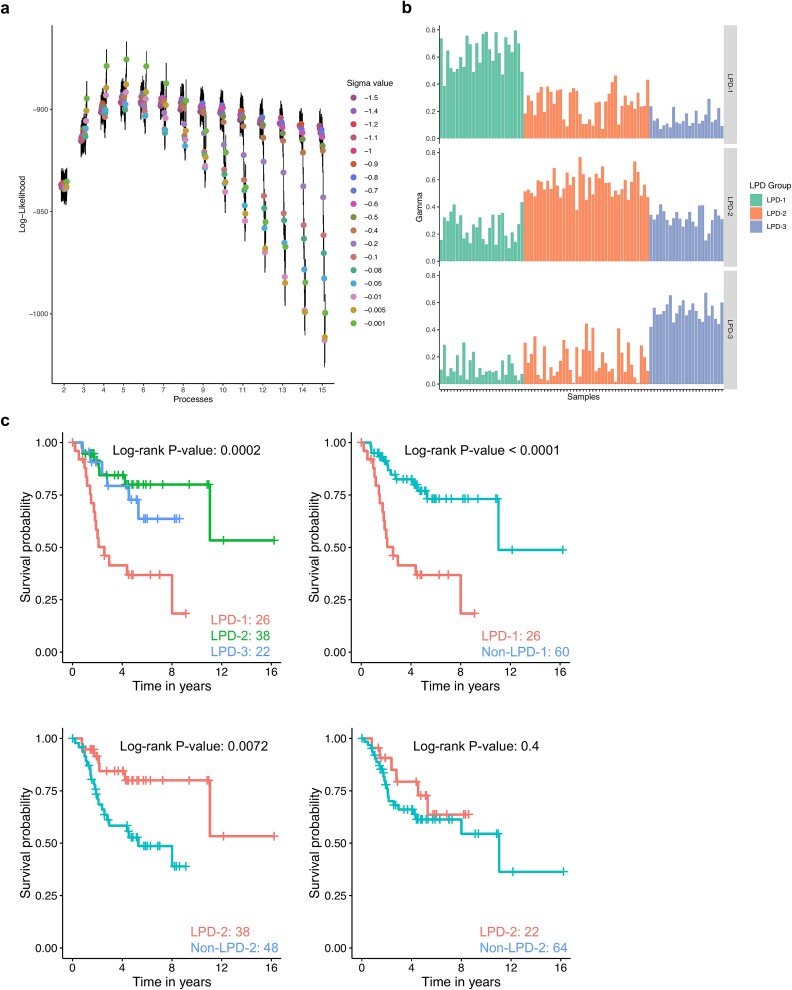
Latent process decomposition model optimization, subtype assignment and clinical outcome. (a) Hyperparameter optimization for the TARGET dataset. LPD assesses the explanatory power of different combinations of sigma values (process spread) and the number of processes. The optimal combination is determined as the point of maximum log-likelihood before the onset of overfitting, visually identified as a plateau in the curves. For the TARGET dataset, the optimal parameters were three processes and a sigma value of −0.0001. (b) Sample assignment to subtypes. Bar plot illustrates sample assignment to the three identified subtypes based on their degree of membership (gamma value). Higher gamma values indicate stronger membership in a specific subtype reflecting the extent to which each subtype captures sample-specific transcriptomic variability. (c) Kaplan–Meier curves illustrate the survival probability over time for each subtype. Pairwise comparisons between subtypes are shown with log-rank *p*-values and sample sizes provided for each comparison.

We analyzed 165 primary osteosarcomas using RNA-seq data from the TARGET osteosarcoma initiative phs000468 plus the GREEN [40], PERRY [41], and SCOTT [42] datasets (Table 1). To expand our analysis, we also included 12 metastatic samples, 5 samples from cell lines, 5 circulating tumor cell samples, and 3 normal bone samples. LPD decomposes the expression profile from each sample into underlying components termed “processes.” By analyzing the relative abundance of these processes within a sample, LPD can catalog complex data and objectively assess the optimal number of processes. This assessment was achieved through hold-out validation and by tuning the sigma hyperparameter that represents the spread of the processes. The optimal parameter combination was identified as the point with the maximum log-likelihood prior to the onset of overfitting (Figure 1a). For the model development dataset (TARGET), three disease subtypes and a sigma value of −0.001 were determined. To ensure robustness, LPD was iterated 100 times with varying random seeds using this parameter combination. From these iterations, 94 showed associations with a poor prognosis (e.g., low overall survival) subtype. The subtype with the survival log-rank close to the mode (*P* = 5.92 × 10^−05^) was selected for further analysis.

### Characterization of the three osteosarcoma molecular subtypes

Each process detected by LPD presented a degree of membership reflecting the extent to which each subtype captured the transcriptomic variability in the samples. We classified the samples according to the process most abundant within them into three groups termed TARGET LPD-1 (*n* = 26), TARGET LPD-2 (*n* = 39), and TARGET LPD-3 (*n* = 23) (Figure 1b). We studied the associations of each group membership with age, sex, ethnicity, vital status, and survival probability. TARGET LPD-1 exhibited distinct clinical characteristics: predominantly female and had a higher mortality rate compared to patients in the other subtypes (*P* < 0.05 for both comparisons; Supplementary File 2; Table 2). While the patients in TARGET LPD-1 appeared to be younger than those in the other subtypes, this difference did not reach statistical significance after Bonferroni correction for multiple comparisons (*P* = 0.18). Kaplan–Meier analysis confirmed a significantly lower overall survival for patients with TARGET LPD-1 (*P* < 0.001; Figure 1c). Cox regression models showed that patients with TARGET LPD-1 had a 1.6-fold increased risk of death when compared to those with TARGET LPD-2 (HR = 1.633, *P* < 0.001) (Supplementary File 2) and a 1.1-fold increased risk when compared to those with TARGET LPD-3 (HR = 1.144, *P* = 0.017) (Supplementary File 2).

**Table 2:** Clinical characteristics of the three disease subtypes.

		LPD-1	LPD-2	LPD-3	*P* value
*n*		26	39	23	
Age	Median	12.5	15	15	0.184
	IQR	10–15.8	13–17.5	12–18	
Sex	Female	17	9	11	0.01
	Male	9	30	12	
Ethnicity	Asian	2	3	2	1.00
	Black/African descent	2	3	2	
	Caucasian/European descent	12	27	13	
Vital status	Alive	10	31	16	0.004
	Dead	16	7	6	

For each subtype, median age and interquartile range (IQR) are provided. Sex, ethnicity, and vital status distributions are shown, along with corresponding *P* values from ANOVA (age) and chi-square tests (sex, ethnicity, vital status). *P* values are Bonferroni-adjusted for multiple comparisons.

### Targetable genes in the three osteosarcoma subtypes

To identify DE transcripts within the three LPD osteosarcoma subtypes, we performed pairwise comparisons. We identified 679 DE transcripts (336 upregulated, 343 downregulated) with an absolute log_2_ fold change exceeding 1.5, an adjusted *P*-value below 0.01 and detection in at least 80% of the runs (Supplementary File 3). GO enrichment analysis revealed 69 significantly altered biological processes associated with these DE transcripts. Extracellular matrix structure pathways were the most overrepresented with 35 genes involved (Supplementary File 4).

### LPD model validation

To validate the model using the data available, we applied the LPD model to the GREEN (*n* = 14), PERRY (*n* = 35), and SCOTT (*n* = 52) datasets. Due to the limited clinical data in the validation datasets, we selected the optimal LPD iteration for each dataset based on its similarity to the poor prognosis TARGET LPD-1 subtype (as determined by gene expression patterns). This iteration identified two subtypes in GREEN (GREEN LPD-1, GREEN LPD-2), three in PERRY (PERRY LPD-1, PERRY LPD-2, PERRY LPD-3), and four in SCOTT (SCOTT LPD-1, SCOTT LPD-2, SCOTT LPD-3, SCOTT LPD-4).

The GREEN subtypes exhibited differences in sample composition. GREEN LPD-1 (*n* = 10) was mostly comprised of primary and metastatic samples (Chi-Square test, *P* = 0.006) (Supplementary File. 2). GREEN LPD-2 (*n* = 4) exclusively consisted of circulating tumor cells. No clinical data were available.

No significant associations were found between PERRY subtypes and age, sex, or vital status. Kaplan–Meier survival curves showed a nonsignificant (*P* = 0.15) trend toward poorer survival for PERRY LPD-1 (*n* = 8) and PERRY LPD-2 (*n* = 14) when compared to PERRY LPD-3 (*n* = 13) (Supplementary File 2).

Significant age differences were observed among SCOTT subtypes (ANOVA, *P* = 0.005). Post hoc Tukey tests revealed that SCOTT LPD-1 (*n* = 13) and SCOTT LPD-3 (*n* = 26) were significantly younger than SCOTT LPD-4 (*n* = 7). SCOTT LPD-2 (*n* = 6) primarily consisted of osteosarcoma cell lines (Chi-square, *P* < 0.001) (Supplementary File 2). SCOTT LPD-1 was the only group without normal bone samples. No survival data were available.

### Shared molecular mechanisms between the “poor prognosis” subtypes

**Figure 2:**
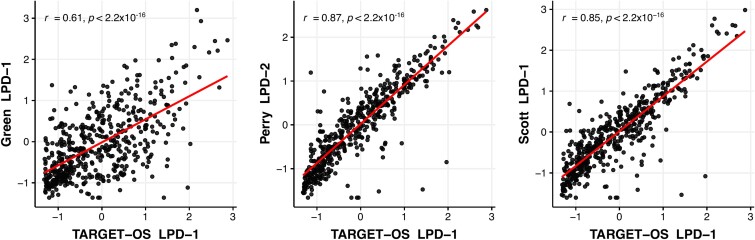
Correlation of gene expression profiles between poor prognosis TARGET LPD-1 and corresponding subtypes. Scatter plots comparing the expression levels of the top 500 most variable transcripts across the entire TARGET dataset between TARGET LPD-1 and the corresponding most similar subtypes from the GREEN (GREEN LPD-1), PERRY (PERRY LPD-2), and SCOTT (SCOTT LPD-1) datasets. Trend lines and Pearson correlation coefficients (r) with corresponding *P*-values are displayed for each comparison.

**Figure 3:**
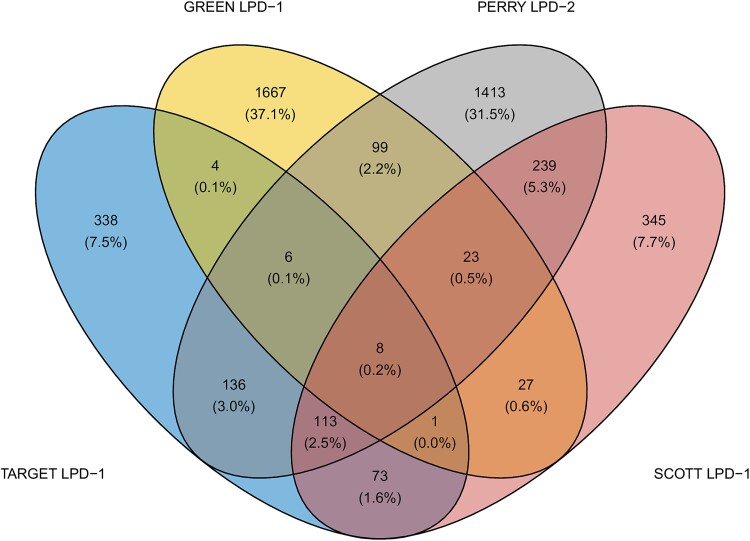
Overlap of DE transcripts. Venn diagram illustrating the overlap of DE transcripts between TARGET LPD-1 and the most closely correlated subtypes from the GREEN, PERRY, and SCOTT datasets. The diagram quantifies the number of DE transcripts in each dataset and identifies eight transcripts shared across all four poor prognoses datasets.

To identify potential shared molecular mechanisms underlying the poor prognosis phenotype, we performed DE analysis and GO enrichment on the disease subtypes most closely resembling TARGET LPD-1. These subtypes were GREEN LPD-1 (Pearson correlation, *P* < 0.001), PERRY LPD-2 (Pearson correlation, *P* < 0.001), and SCOTT LPD-1 (Pearson correlation, *P* < 0.001) (Figure 2). A complete list of DE transcripts and enriched biological processes is presented in Supplementary File 5. We defined a core gene set comprising eight transcripts shared across all datasets (three upregulated: *ANGPT1*, *CGREF1*, *KAZALD1*; five downregulated: *CILP*, *COL25A1*, *MASP1*, *SDK1*, *SEMA5B*) (Figure 3).

### LPD outperforms traditional clustering methods

**Figure 4:**
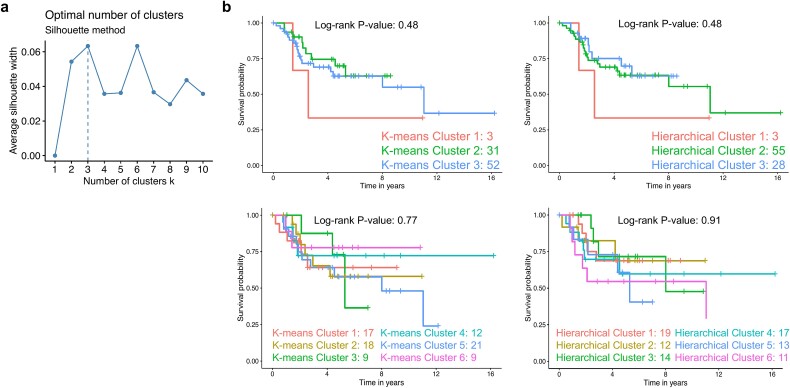
Comparative evaluation of traditional clustering methods. (a) Silhouette analysis to determine the optimal number of clusters for hierarchical and k-means clustering in the TARGET dataset. Three clusters were identified as optimal, with six clusters showing similar performance. (b) Kaplan–Meier survival curves comparing patient survival based on hierarchical and k-means clustering groups using both three and six clusters as suggested by the silhouette analysis. Log-rank test was used to assess statistical significance.

We compared the prognostic capabilities of LPD to the traditional clustering methods hierarchical and k-means using the TARGET dataset. Based on silhouette scores (Figure 4a), the optimal number of clusters for both hierarchical and k-means clustering was three, followed by six. Subsequent Kaplan–Meier survival analyses (Figure 4b) revealed no significant differences in survival between the clusters (*P* > 0.05) indicating that these classical methods failed to identify prognostic subgroups comparable to those identified by LPD.

## Discussion

Bioinformatics approaches applied to cancer transcriptome data have categorized clinically relevant breast cancer subtypes [4]. For “difficult” cancers such as osteosarcoma no associations between molecular profiles, clinical presentation, or survival outcomes are known; therefore, untargeted chemotherapy remains the backbone of treatment. Because of the challenges associated with these difficult cancers (e.g., rarity, heterogeneity, lack of funding, etc.) computational methods need to be adapted and improved to make the best use of the few available samples. We have used a more sophisticated unsupervised Bayesian method termed LPD, which considers individual tumor sample heterogeneity (where previous methods do not). Our results confirmed the emerging prediction that osteosarcoma is not one disease. Using 165 primary osteosarcomas to develop the model, we detected three clinically relevant disease subtypes. One subtype in particular was significantly associated with a poor prognosis, at least, when treated with chemotherapy. In the future, clinical trials should group patients with osteosarcoma according to their subtype (based on gene expression) so that treatment is tailored to their disease. We expect that this new diagnostic labeling followed by stratified treatment will significantly improve osteosarcoma survival; as well as other challenging cancers that might be assessed by LPD.

Our analyses uncovered a core gene set of eight consistently dysregulated transcripts across several osteosarcoma datasets. These genes are promising candidates for biomarker development and therapeutic targeting. Several of these genes have been studied in osteosarcoma including *ANGPT1* upregulation associated with nonmetastatic disease [47] and *CGREF1* overexpression linked to a poor prognosis [48]. *KAZALD1* is overexpressed in osteoblastic osteosarcoma and carcinoma-associated fibroblasts [49, 50] and *CILP* under expression is a known feature of the disease [51]. Although not directly linked to osteosarcoma, *COL25A1* expression has been studied as a potential pancancer prognostic indicator alongside other collagen family genes [52]. *MASP1*, involved in the immune response, lacks an association with osteosarcoma in the literature but might influence the tumor microenvironment. *SDK1* and *SEMA5B* implicated in prostate and kidney cancer, respectively [53, 54], represent novel gene candidates with potential implications for understanding osteosarcoma heterogeneity.

Previous work using an unsupervised machine learning strategy defined a repertoire of independent components describing the transcriptional program of osteosarcoma tumors and tumor microenvironments at diagnosis [34]. Using a 15 gene signature in a cohort of 82 patients, the study discriminated “favourable” and “unfavorable” prognoses, proposing two tumor phenotypes already present at diagnosis and presumed to respond differentially to treatment [34]. Favorable prognosis tumors termed G1 were associated with innate immune expression. Unfavorable prognosis tumors termed G2 were associated with a tumor microenvironment comprising angiogenic, osteoclastic, and adipogenic activities prone to induce metastases [34]. At the clinical level, this G1/G2 framework was consistent with the observed efficacy of tyrosine kinase inhibitors with antiangiogenic activity in relapsed osteosarcoma [55]. The inefficacy of zoledronate in frontline osteosarcoma treatment [56] was thought to be partially linked to its action on the immune system [57]. In the context of the current work, we also observed dysregulation of immune-related transcripts between the detected subtypes. These independent analyses emphasize the future important role of immunotherapies in osteosarcoma treatment.

An important issue for patients diagnosed with osteosarcoma is that the clinical outcome is highly variable. Precise prediction of disease progression at the time of diagnosis is not possible. In some retrospective studies, more than 90% tumor necrosis after induction therapy is associated with increased survival [58]. Around 45% of patients do not achieve this threshold. It is still not possible to predict who is likely to respond to chemotherapy. The limited number of studies investigating drug resistance has not been able to describe refractory disease or predict response [59, 60]. Augmenting cytotoxic regimens has been unsuccessful. The addition of ifosfamide and etoposide to the methotrexate, doxorubicin, and cisplatin (MAP) chemotherapy backbone has not improved survival [61, 62]. The limits of toxicities have been reached, so it is unlikely that progress will be made through trials of drug variations that are “more of the same” [13]. Novel strategies beyond cytotoxic chemotherapy are required to achieve an increase in cure rates [13]. There is an urgent need for the identification of osteosarcoma categories linked to targeted therapies. For breast cancer, machine learning-based clustering of transcriptome data has resulted in a classification system that is used to guide disease management and treatment.

 Data platforms, e.g., The Cancer Genome Atlas, include gene expression, mutation, and methylation data for several cancer types. The relative ease of downloading data from multiple platforms has prompted the development of new computational methods for subclass discovery including the copula-mixed model [63], Bayesian consensus clustering [64], and the iCluster model [65], which can combine data from the different platforms. Within machine learning approaches, such analyses are termed “supervised” or “unsupervised” [66]. In a supervised setting, the objective is to identify transcriptomic variations that predict disease state or are strongly correlated with clinically significant variables [67, 68]. Unsupervised learning typically involves identifying latent substructures in the data that can be used to learn more about disease etiology such as cancer subtypes [69, 70]. Such approaches, however, suffer the problem of sample assignment to a particular cluster or group and the failure to take into consideration the heterogeneous composition of individual samples. These fundamental flaws highlight the need to develop more sophisticated methods similar to LPD that can be applied to multiple platform data [35].

In summary, we have established a novel stratification framework for the analysis of osteosarcoma that has its origins in unsupervised machine learning analyses of transcriptome data that also considers the heterogeneous composition of individual cancer samples. This framework has identified three osteosarcoma disease subtypes. One of the subtypes was found to respond poorly when treated with a MAP chemotherapy backbone. These data will be critical for future diagnostic labeling and sorting patients into groups before clinical trial allocation and administering more effective stratified medicines. In future, we plan to analyze the utility of LPD in managing patients with osteosarcoma including predicting the response to experimental drug treatments. This work will be performed through the assessment of LPD status in the contexts of clinical trials.

### Limitations of the study

Two of the key limitations of the study were the small dataset used for the LPD model development (*n* = 88) and the incomplete clinical data in the validation cohort (*n* = 77). Access to tissue and linked clinical data is particularly challenging for osteosarcoma due to the rarity of cases, limited biopsy material, and the extensive chemotherapy-related damage present in posttreatment samples. These problems paired with also needing to analyze publicly available RNA-seq data where the library preparation methods and sequencing platforms are not dissimilar makes more sophisticated analyses such as LPD more challenging. These types of studies typically require thousands of samples with associated (and consistent) clinical data. Despite the issues faced, the LPD approach was robust, with the biologically defined subgroups appearing across four different datasets. As with all machine learning methods, their output significantly improves and refines with the addition of more samples. The recent release of new Europe-wide clinical guidelines for improving bone sarcoma biological sample and associated clinical data collection [3] means that it is reasonable to presume that a second version of the LPD model, perhaps performed in ~5 years’ time, might reveal even more molecular subtypes.

**Key Points**

- There is a current lack of a machine learning solution that can assess a patient’s rare and difficult cancer (e.g., osteosarcoma) subtype based on RNA-seq data.
- We have developed a Bayesian unsupervised clustering model for three osteosarcoma disease subtypes. The models are structured to output gene expression and functional analysis data that can be used for stratifying treatment beyond that of untargeted chemotherapy.
- This new machine learning algorithm should be used to classify patients with rare cancers such as osteosarcoma. The new tool could help clinicians and clinical trialists to predict the response to new and experimental drugs.

## Supplementary Material

Suppl_File_1_bbae665

Suppl_File_2_bbae665

Suppl_File_3_bbae665

Suppl_File_4_bbae665

Suppl_File_5_bbae665

## Data Availability

This study used publicly available data available for download from the original citations used in the text.
